# Cetuximab–Toxin Conjugate and NPe6 with Light Enhanced Cytotoxic Effects in Head and Neck Squamous Cell Carcinoma In Vitro

**DOI:** 10.3390/biomedicines12050973

**Published:** 2024-04-29

**Authors:** Noriko Komatsu, Azuma Kosai, Mikako Kuroda, Takao Hamakubo, Takahiro Abe

**Affiliations:** 1 Department of Oral and Maxillofacial Surgery of Dentistry, Kanagawa Dental University, Yokosuka 238-8570, Japan; n.komatsu@kdu.ac.jp (N.K.); a.kosai@kdu.ac.jp (A.K.); mikakoc782@gmail.com (M.K.); 2 PhotoQ3 Inc., Tokyo 102-0084, Japan; t.hamakubo@photoq3.com

**Keywords:** endosomal escape, immunotoxin, mono-L-aspartylchlorine6, photodynamic therapy (PDT), iTAP, epidermal growth factor receptor, head and neck squamous cell carcinoma

## Abstract

Background: Photodynamic therapy (PDT) is a cancer-targeted treatment that uses a photosensitizer (PS) and irradiation of a specific wavelength to exert cytotoxic effects. To enhance the antitumor effect against head and neck squamous cell carcinoma (HNSCC), we developed a new phototherapy, intelligent targeted antibody phototherapy (iTAP). This treatment uses a combination of immunotoxin (IT) and a PS for PDT and light irradiation. In our prior study, we demonstrated that an immunotoxin (IT) consisting of an anti-ROBO1 antibody conjugated to saporin, when used in combination with the photosensitizer (PS) disulfonated aluminum phthalocyanine (AlPcS2a) and irradiated with light at the appropriate wavelength, resulted in increased cytotoxicity against head and neck squamous cell carcinoma (HNSCC) cells. ROBO1 is a receptor known to be involved in the progression of cancer. In this study, we newly investigate the iTAP targeting epidermal growth factor receptor (EGFR) which is widely used as a therapeutic target for HNSCC. Methods: We checked the expression of EGFR in HNSCC cell lines, SAS, HO-1-u-1, Sa3, and HSQ-89. We analyzed the cytotoxicity of saporin-conjugated anti-EGFR antibody (cetuximab) (IT-Cmab), mono-L-aspartyl chlorin e6 (NPe6, talaporfin sodium), and light (664 nm) irradiation (i.e., iTAP) in SAS, HO-1-u-1, Sa3, and HSQ-89 cells. Results: EGFR was expressed highly in Sa3, moderately in HO-1-u-1, SAS, and nearly not in HSQ-89. Cmab alone or IT-Cmab alone did not show cytotoxic effects in Sa3, HO-1-u-1, and HSQ-89 cells, which have moderate or low expression levels of EGFR protein. However, the iTAP method enhanced the cytotoxicity of IT-Cmab by the photodynamic effect in Sa3 and HO-1-u-1 cells, which have moderate levels of EGFR expression. Conclusion: Our study is the first to report on the iTAP method using IT-Cmab and NPe6 for HNSCC. The cytotoxic effects are enhanced in cell lines with moderate levels of EGFR protein expression, but not in nonexpressing cell lines, which is expected to expand the range of therapeutic windows and potentially reduce complications.

## 1. Introduction

Head and neck cancer (HNSCC) accounts for approximately 3% of all cancers and tends to increase [1]. The head and neck region is responsible for many functions such as eating, speaking, and breathing [2]. One significant challenge is that traditional treatment methods, including surgical procedures, chemotherapeutic agents, and radiation therapy, often lead to persistent functional impairments. These impairments can manifest as difficulties with eating, speech, and physical appearance, ultimately compromising the patient’s quality of life (QOL) [2]. These treatment-related complications should be minimized. Antibody drugs are one of the treatments with fewer complications. Several antibody-based therapies, including cetuximab, nivolumab, and pembrolizumab, are currently employed in the treatment of head and neck cancer. A recent development in this field is the approval of Cetuximab sarotalocan sodium (Akalux^®^), a novel drug that conjugates cetuximab with a dye known as IR-700. This innovative combination therapy has been approved for the treatment of head and neck cancer that is either unresectable, locally advanced, or locally recurrent [3,4,5,6]. However, for antibody drugs to exhibit high antitumor effects, high levels of expression of target factors are required. Therefore, it is desirable to develop drugs and treatments that exert cytotoxic effects with limited target expression levels.

We have studied the development of novel treatments to minimize treatment-related complications and increase antitumor effects. In our previous study, we discovered that an immunotoxin (IT) composed of an anti-ROBO1 antibody linked to saporin, when administered in conjunction with the photosensitizer (PS) disulfonated aluminum phthalocyanine (AlPcS2a) and exposed to light at a wavelength of 680 nm, potentiates cytotoxicity in head and neck squamous cell carcinoma (HNSCC) cells. ROBO1 is a receptor known to play a role in the progression of cancer. [7,8]. We have developed a novel method using a PS for PDT, mono-L-aspartylchlorine6 (NPe6), and named this therapeutic method “intelligent targeted antibody phototherapy” (iTAP) [9]. We first reported saporin-conjugated anti-epidermal growth factor receptor (EGFR) antibody target EGFR (cetuximab) (IT-Cmab), NPe6, and light (664 nm) irradiation (i.e., iTAP) in lung cancer cells in 2022. IT is made by conjugating saporin to an anti-EGFR antibody. Saporin, a toxic protein derived from the Saponaria officinalis plant, belongs to the category of type 1 ribosome-inactivating proteins (RIPs). Its cytotoxic effects are triggered only after it is released from endosomes and enters the cytosol of the cell [10,11]. PDT has been used for cancer-targeted treatment. A tumor-affinity photosensitizer (PS) and light of a specific wavelength are used. First, PS localizes to the target cell and/or tissue. Second, the specific wavelength excites the PS. The excited PS transfers its energy to nearby molecular oxygen, which converts it to highly reactive singlet oxygen and reactive oxygen species (ROS), which damage tumor cells [12,13,14,15]. 

In contrast to conventional treatments that have many complications, PDT is minimally invasive and it is possible for it to be performed repeatedly with no cumulative toxicity [15,16]. PS accumulates on the cell membrane, whereafter IT-Cmab specifically binds to EGFR expressed on the cancer cell membrane. Both of them are then endocytosed to the endosome. During subsequent irradiation with the specific wavelength of PS, the photodynamic effect causes the endosome to rupture, enabling the endosomal escape of IT (i.e., iTAP) [7,8,9]. It was inferred that saporin accumulates at high concentrations in the cytoplasm of cancer cells with high expression of EGFR, and specifically leads to cell death [7,8,9]. 

Therefore, iTAP has a higher antitumor effect than PDT, antibody, or IT alone [7,8,9]. To examine the possibility of clinical application of iTAP, we used an anti-epidermal growth factor receptor (EGFR) antibody immunotoxin (IT-cetuximab, IT-Cmab) that targets EGFR, which is widely used for the treatment of HNSCC. Mono-L-aspartyl chlorin e6 (NPe6, talaporfin sodium) is used in iTAP, which excites at approximately 664 nm, as a photosensitizer. Photodynamic therapy (PDT) with NPe6 already has a clinical record of being used for treatments on early-stage non-small-cell lung cancer, esophageal cancer, malignant brain tumors, etc. [17,18,19]. Our study verifies the cytotoxic effects of iTAP using IT-Cmab and PDT (NPe6, 664 nm) on HNSCC.

## 2. Materials and Methods

### 2.1. Cell Culture

The cell lines we used are all derived from human HNSCCs. Sa3 (derived from the upper gingiva, RCB0980), HO-1-u-1 (derived from the floor of the mouth, RCB2102), SAS (derived from the tongue, RCB1974), and HSQ-89 (derived from the maxillary sinus, RCB0789) were purchased from RIKEN (Saitama, Japan) [20,21,22,23]. Sa3 cells were cultured in Basic Minimum Essential Medium (BME) supplemented with 20% newborn bovine serum (NBS). HO-1-u-1 and SAS cells were cultured in RPMI1640 supplemented with 10% FBS. HSQ-89 cells were cultured in Dulbecco’s Modified Eagle Medium (DMEM) supplemented with 10% fetal bovine serum (FBS).

### 2.2. Database Analysis

We performed expression analysis of EGFR using Cancer Dependency Map Portal (RRID:SCR_017655), a DepMap 23Q4 Public dataset (https://depmap.org/portal/interactive/, accessed on 1 March 2024). 

### 2.3. Reverse Transcription Real-Time PCR

RNA was extracted from confluent cell cultures using TRIzol (Invitrogen, MA, USA) and purified with NucleoSpin RNA mini (Macherey-Nagel, Düren, Germany). For real-time PCR analysis, 500 ng of total RNA was reverse-transcribed into first-strand cDNA by using ReverTra Ace (Toyobo, Osaka, Japan) and random primers. PCR amplification was performed in a reaction volume of 20 µL containing 1 µL of 10× diluted cDNA and 10 µL KOD SYBR qPCR Master Mix (Toyobo, Osaka, Japan) using qTOWER3G (analytikjena, Jena, Germany). The PCR primers used in this study were designed to amplify the coding region of the *EGFR* gene across the most common variants, based on sequences available in GenBank (accession nos. NM_005228, NM_001346898, NM_201282, NM_201283, NM_201283). Forward primer: 5′-AAGGAGCTGCCCATGAGAAA-3′; reverse primer: 5′-CAGGTGGCACCAAAGCTGTA-3′; product length 476 bp. *GAPDH* was chosen as the reference gene (GenBank accession no. NM_002046), forward primer: 5′-AGTCAGCCGCATCTTCTTTTGC-3′; reverse primer: 5′-AGCATCGCCCCACTTGATTTTG-3′; product length 323 bp. Expression of *EGFR* was normalized against *GAPDH* across cell lines using the ΔCT method [24].

### 2.4. Western Blot Analysis

Confluent cells were lysed in RIPA buffer containing protease inhibitors and phosphatase inhibitors (both Nacalai Tesque, Kyoto, Japan). The lysates were centrifuged at 10,000× *g* at 4 °C for 10 min, and the supernatant containing the protein was collected. Protein concentration was determined using the Bradford assay (Takara Bio, Kusatsu, Japan) according to the manufacturer’s instructions. Equal amounts of protein (2 µg) from each sample were mixed with 2× Laemmli buffer (Biorad, CA, USA), heated at 95 degrees C for 5 min, and then loaded onto 4–20% SDS-polyacrylamide gels (Biorad, CA, USA). Electrophoresis was performed at 200 V until the desired resolution was achieved. Proteins were transferred from the gel to PVDF membrane (ATTO, Taito, Japan) using semi-dry transfer at 25 V for 20 min [25]. The efficiency of transfer was verified with Ponceau S staining (Beacle, Kyoto, Japan). Membranes were blocked with Bullet Blocking One (Nacalai Tesque, Kyoto, Japan) for 5 min at room temperature to prevent nonspecific binding. After blocking, the membranes were incubated with primary rabbit polyclonal antibody against EGFR (dilution 1/1000, HPA018530, Atlas Antibodies, Stockholm, Sweden) and GAPDH (dilution 1/5000, 10494-1-AP, Proteintech, Rosemont, IL, USA) overnight at 4 °C. Following primary antibody incubation, membranes were washed 3 times for 3 min each with TBS-T and then incubated with HRP-conjugated goat anti-rabbit secondary antibody (dilution 1/2000, Proteintech, Rosemont, IL, USA) for 1 h at room temperature. After washing the membrane 3 times for 3 min each with TBS-T, protein bands were visualized by ECL, Chemi-Lumi One (Nacalai Tesque, Kyoto, Japan), according to the manufacturer’s instructions. The signal was detected using Luminograph I (ATTO, Taito, Japan).

### 2.5. Immunotoxin

A saporin-conjugated anti-EGFR antibody (Cmab), hereafter called IT-Cmab, was prepared as follows: biotinylated Cmab was purified by mixing cetuximab with EZ-LINK sulfo-NHS-LC-biotin (Thermo Fisher Scientific, Waltham, MA, USA) at a 1:20 molar ratio using PD-10 (GE Healthcare Life Sciences, Piscataway, NJ, USA), as previously reported [3,4,6]. Next, biotinylated Cmab and streptavidin-saporin (Biotin-Z Internalization Kit [KIT-27-Z]) (Advanced Targeting Systems, Carlsbad, CA, USA) were mixed in approximately equivalent amounts and allowed to react at room temperature for 30 min to obtain IT-Cmab, as previously reported [9].

### 2.6. Cytotoxicity Assay of IT-Cmab and Cmab

Cells were seeded at 5.0 × 10^3^ cells (SAS), and 2.0 × 10^4^ cells (Sa3, HO-1-u-1, HSQ-89) per well in 96-well plates and cultured for 48 h at 37 °C. They were exposed to various concentrations (1.34 pM~4.2 nM) of either IT-Cmab or Cmab. After 72 h, cell viability was assessed with a cell counting kit-8 (CCK-8 kit, Dojindo Laboratories, Kumamoto, Japan). Cell viability was calculated, as previously reported [7,10]. CCK-8 uses WST-8 as a chromogenic substrate. WST-8 is reduced by intracellular dehydrogenase to produce water-soluble formazan. The number of living cells is counted by directly measuring the absorbance of this formazan at 450 nm. Cell viability was calculated using the following formula: Cell viability (%) = (a − c)/(b − c) × 100 a: the absorbance value of each sample, b: the absorbance value for the IT free sample, c: the absorbance value of the blank sample (medium only) [7,10,26]. The mean ± SD of the cell viability values were calculated from 3 independent experiments and plotted on the graph versus the IT concentration. 

The half-maximal inhibitory concentrations (IC_50_) were obtained from the sigmoid curve using the curve-fitting tool of the version 1.51v ImageJ software [7,10].

### 2.7. Light Sources

The illumination system for exposing the cells was developed in-house. We used a custom setup where half of a 96-well plate received light from both sides. The light source was 670 nm LEDs (SMBB670D-1100, USHIO Optical Semiconductors, Tokyo, Japan), which were homogenized and expanded using a Köhler integrator configuration [27,28]. This arrangement created a top-hat profile uniformly lit hexagonal area (75.4 cm^2^) with an intensity of 9.28 mW/cm^2^. The LEDs operated on a stable DC power supply (WANPTEK, Shenzen, PRC). Irradiance was measured with a power meter LP10 (SANWA Electric, Tokyo, Japan) and corrected for wavelength. The duration of illumination was controlled using an Arduino Nano (Arduino, Ivrea, Italy).

### 2.8. Photosensitizer

Mono-L-aspartyl chlorin e6 (NPe6, talaporfin sodium, Laserphyrin) was purchased from Meiji Seika Pharma (Tokyo, Japan). NPe6 was adjusted to 2 mg/mL by DMSO by using ultrasound. NPe6 has a maximum absorption peak at 407 nm and a second peak at 664 nm [15,29].

### 2.9. Cytotoxicity Assay of NPe6

SAS was seeded at 5.0 × 10^3^ cells. Sa3, HO-1-u-1, and HSQ-89 were seeded at 2.0 × 10^4^ cells per well in 96-well plates and cultured for 48 h at 37 °C. They were exposed to various concentrations (0.032–100 μM (SAS), 0.032–100, 250 μM (Sa3, HO-1-u-1, HSQ-89)) of NPe6 and cultured at 37 °C. Then, 24 h after administration of these drugs, the culture medium was changed to drug-free medium. The cells were then irradiated from an LED lamp (670 nm) for 39.5 min (9.28 mW/cm^2^, 22 J/cm^2^). After 48 h, cell viability was assessed with a CCK-8 kit. Cell viability and IC_50_ were determined, as previously reported [7,10]. The optimum concentration of NPe6 was independently determined for each cell line. Based on the results, we decided on final concentrations of NPe6, which were 0.8 µM (SAS), 20 µM (Sa3, HO-1-u-1), and 16 µM (HSQ-89), to use in iTAP.

### 2.10. Cytotoxicity Assay of iTAP Using IT-Cmab, NPe6, and the Exciting Wavelength

Cells were seeded and cultured the same as in the cytotoxicity assay. They were exposed to various concentrations (0.0013–4.2 nM) of IT-Cmab and the predetermined optimum concentration of NPe6 at 37 °C. Then, 24 h after administration of these drugs, the culture medium was changed to a drug-free medium. The cells were then irradiated from an LED lamp (670 nm) for 39.5 min (9.28 mW/cm^2^, 22 J/cm^2^). After 48 h, cell viability was assessed as previously described [7,10]. 

### 2.11. Comparison with Cmab with PDT and IT-Cmab with PDT (iTAP) about Cytotoxicity Assay

Cells were seeded and cultured the same as in the cytotoxicity assay. They were exposed to various concentrations (0.0013–4.2 nM) of Cmab or IT-Cmab and the predetermined optimum concentration of NPe6 at 37 °C. Then, 24 h after administration of these drugs, the culture medium was changed to the drug-free medium. The cells were then irradiated from an LED lamp (670 nm) for 39.5 min (9.28 mW/cm^2^, 22 J/cm^2^). After 48 h, cell viability was assessed, as previously described [7,10].

### 2.12. Statistical Analysis

Data are shown as mean ± SD. Statistical evaluation was performed using analysis of variance (ANOVA) followed by Tukey’s honest significant differences test, as previously reported [7,10]. A *p*-value of <0.05 was taken to be statistically significant.

## 3. Results

### 3.1. Expression of EGFR in Various HNSCC Cells

**Figure 1:**
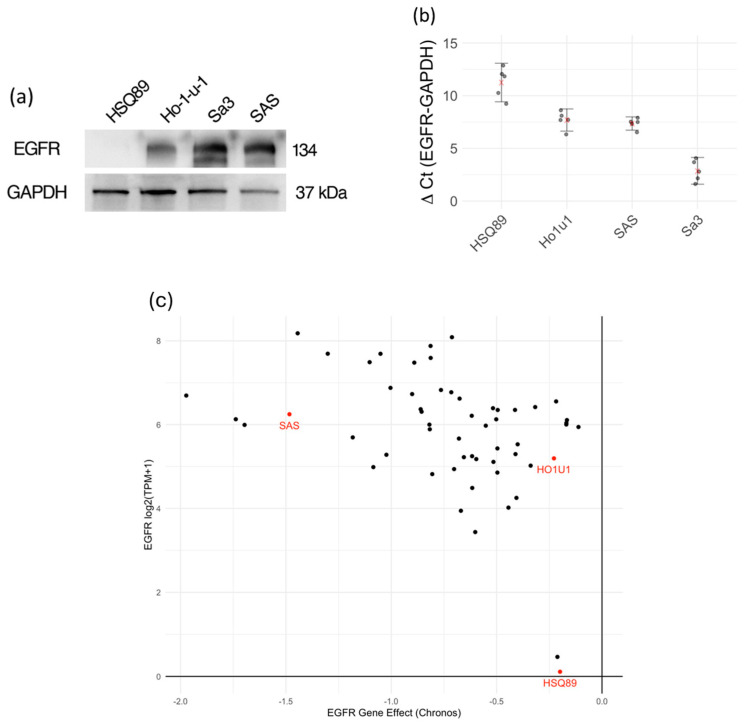
The expression levels of the EGFR protein and mRNA in each HNSCC cell line. WB of EGFR and GAPDH. EGFR protein was detected in Sa3, HO-1-u-1, and SAS, whereas HSQ-89 expression was nondetectable (**a**). *EGFR* mRNA expression measured by real − time PCR. Higher ΔCt indicates lower *EGFR* mRNA expression. mRNA expression of *EGFR* was moderate in HO-1-u-1 and SAS, and high in Sa3. HSQ-89 showed a very low expression of *EGFR*. The red x− mark indicates the mean, and error bars show 95% confidence interval of *n* = 5 experiments (**b**). In the DepMap 23Q4 public dataset (https://doi.org/10.25452/figshare.plus.24667905.v2, accessed on 1 March 2024), the effect of *EGFR* gene knockout is shown in CHRONOS score against *EGFR* mRNA expression in HNSCC cell lines. Sa3 is absent from the dataset (**c**).

The expression levels of EGFR proteins on the surface of each cell were estimated by Western blot. The EGFR protein band was detected in Sa3, HO-1-u-1, and SAS cells at approximately 134 kDa (Figure 1a). The protein level of the EGFR correlated well with the mRNA expression in each of the cell lines (Figure 1b). The EGFR expression level for each cell line was consistent with RNA-seq results in the DepMap database (Figure 1c). 

### 3.2. Cytotoxicity Assay

**Figure 2:**
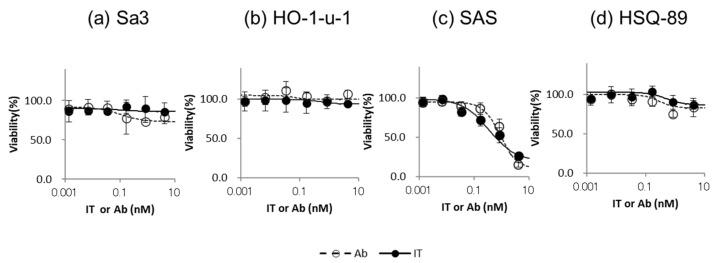
Cytotoxicity of IT-Cmab or Cmab on each HNSCC cell line. We analyzed the cytotoxicity of IT-Cmab or Cmab in Sa3 (**a**), HO-1-u-1 (**b**), SAS (**c**), and HSQ-89 (**d**) cells. The cytotoxicity of IT-Cmab or Cmab was dose-dependent in SAS (**c**) cells. However, cytotoxicity was not significantly different between Cmab and IT-Cmab (**c**). Cytotoxicity of IT-Cmab or Cmab was not observed in Sa3, Ho-1-u-1, and HSQ-89 (**a**,**b**,**d**) cells.

First, the cytotoxicity of Cmab and IT-Cmab on SAS with moderate expression of EGFR protein was dose-dependent. Cytotoxicity was not significantly different between Cmab and IT-Cmab (*p* > 0.05) (Figure 2a–d). IC_50_ of IT-Cmab was approximately 0.07 nM in SAS (Figure 2c). Cmab or IT-Cmab was ineffective in Sa3, HO-1-u-1, and HSQ-89 (Figure 2a,b,d) with low to high expression of EGFR protein. We considered that Cmab and IT-Cmab internalization is insufficient in target cells in Sa3, HO-1-u-1, and HSQ-89. 

**Figure 3:**
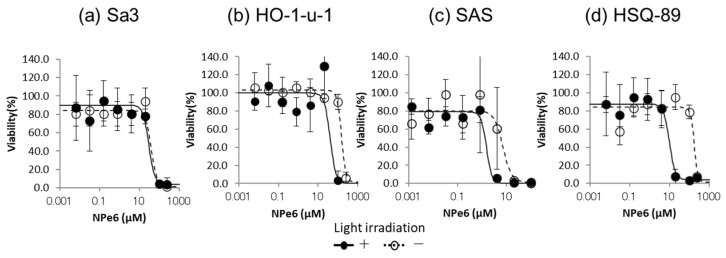
Cytotoxicity of NPe6 and light irradiation on each HNSCC cell line. We examined the cytotoxicity of NPe6 and light irradiation to determine the appropriate concentration for each cell line. The cytotoxicity of Npe6 was dose-dependent in Sa3 (**a**), Ho-1-u-1 (**b**), SAS (**c**), and HSQ-89 (**d**) cells. The maximum concentrations of NPe6 that were nonlethal with NPe6 and light irradiation were 20 μM for Sa3 and HO-1-u-1 (**a**,**b**), 0.8 μM for SAS (**c**), and 4 μM for HSQ-89 (**d**) cells.

Second, to increase the endosomal escape of IT-Cmab, we used the reaction of NPe6 and the excitation wavelength, which disrupt endosomes caused by the generation of ROS. Next, we examined the cytotoxicity of NPe6 itself to determine the appropriate concentration for each cell line. The results showed that the effect of NPe6 on cell toxicity varied depending on the cell line and the presence or absence of irradiation. In Sa3, there was no significant difference in NPe6 cytotoxicity between irradiated and control cells (*p* > 0.05) (Figure 3a). However, significant differences were observed in the other three cell lines at specific NPe6 concentrations when compared with or without irradiation: 100 µM in HO-1-u-1 (*p* < 0.05) (Figure 3b), 0.16 µM in SAS (*p* < 0.05) (Figure 3c), and 20 and 100 µM in HSQ-89 (*p* < 0.05) (Figure 3d). The maximum nonlethal concentrations of NPe6 with irradiation were 20 μM for Sa3/HO-1-u-1, 0.8 μM for SAS, and 4 μM for HSQ-89 (Figure 3a–d). Based on the above results, these concentrations of NPe6 were used in the following cytotoxicity assays. 

**Figure 4:**
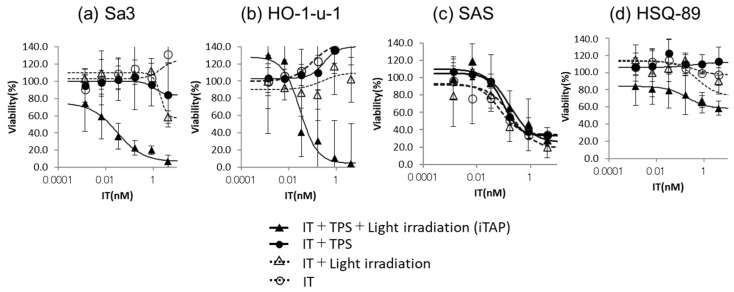
Cytotoxicity assay of iTAP by IT-Cmab, NPe6, and light irradiation. We analyzed the cytotoxicity of IT-Cmab with PDT (iTAP) in Sa3 (**a**), Ho-1-u-1 (**b**), SAS (**c**), and HSQ-89 (**d**) cells. The cytotoxicity of IT-Cmab was neither additive nor synergistic by iTAP in SAS (**c**). Cytotoxicity of IT-Cmab was enhanced by iTAP in Sa3 (**a**) and HO-1-u-1 (**b**) cells. The cytotoxic effects were dose-dependently significant (ANOVA), and the IC_50_ of IT-Cmab was approximately 0.03 nM in Sa3 (**a**) and 0.05 nM in HO-1-u-1 (**b**). The cytotoxicity of IT-Cmab was not enhanced by iTAP in HSQ-89 (**d**).

Third, Sa3 and HO-1-u-1, which have high and moderate expression levels of EGFR protein, exhibited enhanced cytotoxic effects using IT-Cmab with PDT (iTAP) (Figure 4a,b). The cytotoxic effects were dose-dependently significant (ANOVA), and the IC_50_ of IT-Cmab was approximately 0.03 nM in Sa3 and 0.05 nM in HO-1-u-1 (Figure 4a,b). IT-Cmab with PDT (iTAP) demonstrated a significant difference in Sa3 cells, at IT concentrations of 0.16 nM or higher (*p* > 0.05) (Figure 4a). Likewise, in HO-1-u-1 cells, iTAP exhibited a significant difference at IT concentrations of 0.0067 nM or higher (*p* < 0.05) (Figure 4b). SAS, which has moderate expression level of EGFR protein, showed no additive or synergistic effects of cytotoxicity using IT-Cmab with PDT (iTAP) (Figure 4c) (*p* > 0.05). HSQ-89, which does not express EGFR protein, showed no cytotoxic effects using IT-Cmab with PDT (iTAP) (Figure 4d). iTAP showed a significant difference compared to the others at IT concentration of only 4.2 nM in HSQ-89 (*p* < 0.05) (Figure 4d).

**Figure 5:**
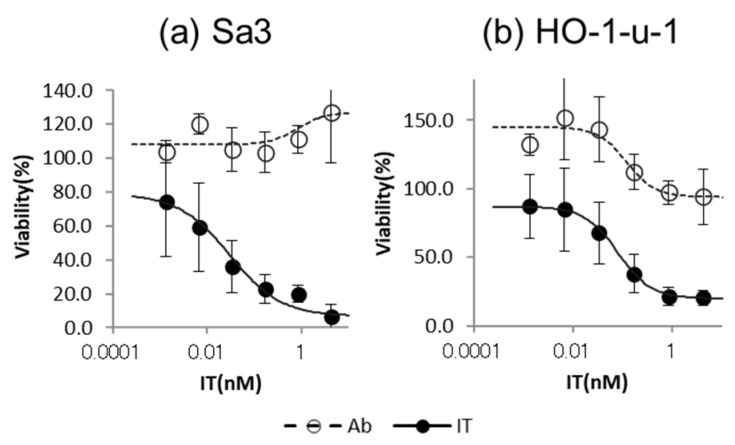
Cell viability of IT-EGFR + PS + illumination compared with anti EGFR antibody + PS + illumination treated in each cell line. We compared the cytotoxicity of Cmab with PDT and IT-Cmab with PDT (iTAP) in Sa3 and HO-1-u-1 cells. The cytotoxicity of Cmab with PDT has no effect on Sa3 and HO-1-u-1 (**a**,**b**). On the other hand, the cytotoxicity of IT-Cmab with PDT (iTAP) was the IT dose-dependent in Sa3 and HO-1-u-1 cells (**a**,**b**).

Finally, we compared the cytotoxic effects of Cmab with PDT and IT-Cmab with PDT (iTAP) in Sa3 and HO-1-u-1. The use of Cmab with PDT had no cytotoxic effects (Figure 5a,b). IT-Cmab with PDT (iTAP) showed a significant difference compared to Cmab with PDT at antibody concentration of 0.0067 nM or greater in Sa3 (*p* < 0.05) (Figure 5a). IT-Cmab with PDT (iTAP) showed a significant difference compared to Cmab with PDT at antibody concentrations of 4.2, 0.8, and 0.0336 nM but not 0.16, 0.0067, and 0.0013 nM in HO-1-u-1 (*p* < 0.05) (Figure 5b). On the other hand, the use of IT-Cmab with PDT (iTAP) has cytotoxicity in Sa3 and HO-1-u-1 (Figure 5a,b).

## 4. Discussion

The cytotoxic effects of antibodies are anticipated to be specific to cancer cells and spare normal cells. To fulfill this, there must be a sufficient difference in the expression levels of target antigen between cancer cells and normal cells [30]. As a result, high-level expression of the antigen is required and therapeutic targets are limited.

In database analysis of EGFR expression and dependency (Figure 1c), SAS shows moderate EGFR protein expression and a low survival rate when EGFR is knocked out (highly EGFR-dependent). In our experiments, cell viability decreased in a concentration-dependent manner with IT-Cmab or Cmab alone in SAS (Figure 2c). On the other hand, there was no enhancement of the decrease in cell viability when we used the iTAP method in SAS (Figure 4c). HO-1-u-1 shows a moderate level of EGFR protein expression (Figure 1a) and has a high survival rate even after being knocked out (low EGFR dependence) (Figure 1c). In HO-1-u-1 cells, cell death was not observed with IT-Cmab or Cmab alone (Figure 2b). However, the application of the iTAP method showed a significant decrease in the cell viability in HO-1-u-1 (Figure 4b). Sa3 has a high level of EGFR protein expression (Figure 1a); however, due to its absence in the DepMap dataset, the effect of EGFR knockout is unknown. In Sa3, cell death was not observed with IT-Cmab or Cmab alone. The iTAP method showed a significant decrease in the cell viability in Sa3 (Figure 2a). HSQ-89 has a low level of EGFR protein expression (Figure 1a) and a high survival rate, even after being knocked out (low EGFR dependence) (Figure 1c). Cell death was not observed with HSQ-89 using IT-Cmab or Cmab alone (Figure 2d). There was no decrease in cell viability, even when using the iTAP method in HSQ-89cells (Figure 4d). SAS exhibits moderate EGFR expression while being extremely sensitive to EGFR knockout (Figure 1c). We hypothesize that iTAP does not show improvement over plain cetuximab since SAS is highly sensitive to cetuximab. On the other hand, HO-1-u-1 exhibits moderate EGFR expression while being not sensitive to EGFR knockout (Figure 1c). 

It is noteworthy that SAS and HO-1-u-1, which have the same moderate EGFR expression level, exhibited different cytotoxic effects using the iTAP method. We believe that this is largely due to the strength of the effect of EGFR signaling blockade. In other words, even if the expression level of EGFR is moderate, if the effect of EGFR knockout is too strong, it is presumed that IT alone can proceed to endosomal escape and exert a cytotoxic effect. Therefore, the cytotoxic effect of the iTAP method is easily exerted. The iTAP method is considered to be suitable for tumors with moderate or higher expression of EGFR and for which knockout of EGFR is less effective. Furthermore, IT-Cmab with PDT showed a cytotoxic effect, but Cmab with PDT did not (Figure 5a,b). In other words, assuming that Cmab and IT-Cmab similarly cause endosomal escape, it is inferred that saporin induces cell death. Therefore, it has been shown that the iTAP method, which uses a combination of IT, in which a toxin is bound to an antibody, and PDT, which uses light and PS, is effective in enhancing the cytotoxic effect.

Based on the above, we demonstrated the effectiveness of the iTAP method (using IT-Cmab and NPe6 with light) in HNSCC cells with moderate EGFR protein expression and low EGFR dependence. In this study, we first report the iTAP method, which enhanced the antitumor effect in HNSCC cells. 

Hamakubo et al. demonstrated the efficacy of IT-Cmab and NPe6 with light (iTAP) in lung cancer. The fluorescence intensity of IT-Cmab and NPe6 with light (iTAP) was significantly higher than that of other conditions. In other words, NPe6 and the specific wavelength enhanced the cytotoxic effect of IT-Cmab by internalizing it into the cytoplasm via endosomal escape. It was inferred that saporin accumulated at high concentrations in cancer cells with high expression of EGFR, and saporin exerted its enzymatic activity only in specific wavelength to NPe6-irradiated cells in this approach [9]. Similarly, in this study, we presumed that iTAP exerted a cytotoxic effect by internalizing IT-Cmab into the cytoplasm. The antitumor effect of the iTap method is mainly due to the cytotoxicity of saporin released into the cytosol by endosomal escape [9]. 

On the other hand, Kobayashi et al.’s near-infrared photoimmunotherapy (NIR-PIT) is another treatment that combines antibody drugs with phototherapy [3,4,5,6]. NIR-PIT showed high antitumor effects in an international phase III clinical trial (LUZERA-301). The cetuximab sarotalocan sodium (Akalux^®^) used in it was approved for the treatment of unresectable locally advanced progressive and recurrent HNSCC in Japan in 2020. IRdye700DX (IR700)-conjugated antibodies bind to target antigen, which destroy tumor cells by specific wavelength light irradiation. When exposed to light at a specific wavelength, the IR700-conjugated antibody that is bound to its target antigen undergoes a chemical reaction. This reaction quickly disrupts the lipid bilayer of the cell membrane, causing the cell to rupture. As a result, the cytoplasmic contents leak out, triggering an immunogenic form of cell death in vivo [3,6]. Therefore, their study and this study have different mechanisms of cytotoxicity. 

Light-based treatments, including the iTAP method, are easier to apply clinically to head and neck cancers where lesions are located relatively superficially. This is due to the limitation of penetration depth of the irradiated light. PDT using light has already been used to treat patients with light irradiation using devices such as bronchoscopes and endoscopes and is used to treat early-stage lung cancer, early-stage esophageal cancer, and early-stage gastric cancer [17,18,19]. The light irradiation devices used in clinical applications by Kobayashi et al. use a frontal diffuser for relatively superficial cancers, and a cylindrical diffuser that is inserted in the lesion for deep cancers. The effective radius of 690 nm irradiation used in the NIR-PIT cylindrical diffusor is approximately 10 mm. By using multiple cylindrical diffusers that penetrate the lesion, it is possible to achieve a therapeutic effect even on large, deep cancers. We believe that there is a possibility of clinical application of iTAP by applying PDT and Kobayashi et al.’s light irradiation device to iTAP.

Taken together, our results suggest that iTAP enhances antitumor effects more than either conventional therapy (Cmab or PDT) and reduced drug doses and illumination irradiation. The immunotoxin and photodynamic therapy (iTAP) approach demonstrates potent antitumor activity and holds promise as a novel therapeutic strategy for the treatment of head and neck squamous cell carcinoma.

## 5. Conclusions

In our current investigation, we found that the immunotoxin and photodynamic therapy (iTAP) approach, which employs an immunotoxin consisting of cetuximab conjugated to saporin (IT-Cmab), the photosensitizer NPe6, and light activation at the appropriate wavelength, demonstrated superior antitumor efficacy compared to cetuximab (Cmab) alone or traditional photodynamic therapy (PDT). Notably, the iTAP method exhibited potential antitumor effects even in tumors expressing moderate levels of the targeted antigens on cancer cells. iTAP reduces the amount of Cmab used and achieves a higher cytotoxic effect than Cmab. In addition, since endosomal escape of drugs caused by iTAP occurs only in the area irradiated with light, we believe that side effects can be reduced. The iTAP method is flexible in its application to antibodies other than Cmab, and is expected to be developed into treatments in various fields. 

## Data Availability

The data presented in this study are available on request from the corresponding author. The data are not publicly available due to the next paper that has an impact on this study has not been published.
